# Individual and combined roles of malonichrome, ferricrocin, and TAFC siderophores in *Fusarium graminearum* pathogenic and sexual development

**DOI:** 10.3389/fmicb.2014.00759

**Published:** 2015-01-12

**Authors:** Shinichi Oide, Franz Berthiller, Gerlinde Wiesenberger, Gerhard Adam, B. Gillian Turgeon

**Affiliations:** ^1^Plant Pathology and Plant-Microbe Biology Section, School of Integrative Plant Science, Cornell UniversityIthaca, NY, USA; ^2^The Research Institute of Innovative Technology for the Earth (RITE)Kizugawa-Shi, Japan; ^3^Department of Agrobiotechnology (IFA-Tulln), Center for Analytical Chemistry, University of Natural Resources and Life SciencesVienna, Austria; ^4^Department of Applied Genetics and Cell Biology, University of Natural Resources and Life SciencesVienna, Austria

**Keywords:** siderophores, malonichrome, *Fusarium graminearum*, virulence, sexual development, HPLC/MS

## Abstract

Intra- and extracellular iron-chelating siderophores produced by fungal non-ribosomal peptide synthetases have been shown to be involved in reproductive and pathogenic developmental processes and in iron and oxidative stress management. Here we report individual and combined contributions of three of these metabolites to developmental success of the destructive cereal pathogen *Fusarium graminearum*. In previous work, we determined that deletion of the *NPS2* gene, responsible for intracellular siderophore biosynthesis, results in inability to produce sexual spores when mutants of this homothallic ascomycete are selfed. Deletion of the *NPS6* gene, required for extracellular siderophore biosynthesis, does not affect sexual reproduction but results in sensitivity to iron starvation and oxidative stress and leads to reduced virulence to the host. Building on this, we report that double mutants lacking both *NPS2* and *NPS6* are augmented in all collective phenotypes of single deletion strains (i.e., abnormal sexual and pathogenic development, hypersensitivity to oxidative and iron-depletion stress), which suggests overlap of function. Using comparative biochemical analysis of wild-type and mutant strains, we show that *NPS1*, a third gene associated with siderophore biosynthesis, is responsible for biosynthesis of a second extracellular siderophore, malonichrome. *nps1* mutants fail to produce this metabolite. Phenotypic characterization reveals that, although single *nps1* mutants are like wild-type with respect to sexual development, hypersensitivity to ROS and iron-depletion stress, and virulence to the host, triple *nps1nps2nps6* deletion strains, lacking all three siderophores, are even more impaired in these attributes than double *nps2nps6* strains. Thus, combinatorial mutants lacking key iron-associated genes uncovered malonichrome function. The intimate connection between presence/absence of siderophores and resistance/sensitivity to ROS is central to sexual and pathogenic development.

## Introduction

The evolutionary diversity and array of genes in filamentous fungi encoding megaenzymes for biosynthesis of secondary metabolites underscore the importance of these factors in fungal biology. While many secondary metabolites are best known for their favorable (e.g., medicinals) or unfavorable (e.g., toxins) effects on other organisms such as humans and agricultural crops, these attributes are largely corollaries to their principal functions in the fungal cells themselves. For example, intra- and extracellular iron-chelating siderophores produced by non-ribosomal peptide synthetases (NRPSs) are centrally, but differentially, involved in developmental processes.

Iron is an essential component of aerobic metabolism and numerous enzymes have Fe-ions as a cofactor. Yet, intracellular free iron (labile iron), in the presence of hydrogen peroxide or superoxide, generates highly cytotoxic reactive oxygen species (ROS), i.e., hydroxyl radicals, from less toxic hydrogen peroxide H_2_O_2_ through the Haber-Weiss/Fenton reaction (Fe^2+^ + H_2_O_2_ → Fe^3+^ + OH^·^ + OH^−^). Tight regulation of the labile iron pool is thus critical for aerobic organisms. Paradoxically, though essential and abundant in the earth's crust, bio-available iron is limited due to low solubility. To solve this dilemma and increase iron solubility, fungi and bacteria produce siderophores, low-molecular weight organic compounds with strong iron-chelating activity biosynthesized by NRPSs (Lee et al., 2005; Oide et al., 2006).

Most fungal siderophores are of the hydroxamate type, produced through condensation of *N^5^*-acyl-*N*^5^-hydroxy-L-ornithine (AHO) units by dedicated NRPSs. Fungal hydroxamate-type siderophores tend to be one of three types, ferrichrome, coprogen, or fusarinine. Members of the latter two groups are produced simply through condensation of AHO units by the NRPSs encoded by the conserved *NPS6*/*sidD* genes (Oide et al., 2006; Schrettl et al., 2007) while the ferrichrome-type is produced through condensation of AHO units plus amino acids such as glycine, serine, or alanine (Bushley et al., 2008). Genes encoding ferrichrome-type siderophore synthetases are conserved across the fungal kingdom and phylogenetic analyses have provided evidence that they can be divided into two lineages, one corresponding to *Cochliobolus heterostrophus NPS2* homologs and the other to *Aspergillus nidulans sidC* homologs (Bushley et al., 2008). Some species, including *Gibberella zeae* (hereafter, *Fusarium graminearum* http://www.ncbi.nlm.nih.gov/pubmed/23379853), carry orthologs of both lineages in their genomes. The metabolites produced by Nps2 and SidC are indispensable for sexual development in heterothallic *C. heterostrophus*, and homothallic *F. graminearum* and *A. nidulans*, indicating functional conservation of these metabolites (Eisendle et al., 2006; Oide et al., 2007). However, functional divergence/overlap between the metabolites produced by the ferrichrome-type siderophore synthetases with distinct evolutionary histories has not been evaluated in a single species.

In our previous work with *F. graminearum*, we identified two NRPSs, one encoded by the *NPS2* gene and the other by the *NPS6* gene, that are involved in siderophore production (Oide et al., 2006, 2007). Biochemical characterization of *nps2* mutants revealed that the Nps2 protein is responsible for biosynthesis of ferricrocin, which acts as an intracellular iron-capturing metabolite in this species. Deletion of *NPS6*, on the other hand, abolished production of the extracellular siderophore, triacetyl fusarinine C (TAFC) and its derivatives. *nps2* mutants are greatly impaired in sexual development, whereas *nps6* mutants show pleiotropic defects including attenuated virulence to wheat, hypersensitivity to ROS, increased sensitivity to iron depletion and reduced asexual sporulation (Oide et al., 2006). Notably, *nps2* mutants are not affected in any of the characteristics found for *nps6* mutants and *vice versa*. *nps6* mutants are thus wild-type (WT) in terms of sexual reproduction, highlighting distinct contributions of intra- and extracellular siderophores to pathogenic, vegetative, and reproductive development of *F. graminearum*.

In addition to *NPS2* and *NPS6*, we (and others) have noted that the genome of *F. graminearum* carries another *NPS* gene likely involved in siderophore biosynthesis (Oide et al., 2007; Tobiasen et al., 2007; Hansen et al., 2012; Adam et al., 2015; Sieber et al., 2014) *NPS1* is an ortholog (Bushley et al., 2008) of *A. nidulans sidC* whose encoded NRPS accounts for ferricrocin synthesis in this species (Eisendle et al., 2006). Our previous attempt to identify the metabolite biosynthesized by Nps1 was unsuccessful, and phenotypic characterization of *nps1* mutants failed to link Nps1 to iron metabolism (Oide et al., 2007). Much earlier work described a siderophore named malonichrome that is produced by a *Fusarium roseum* strain (ATCC 12822) (Emery, 1980). The structure established for malonichrome is that of a ferrichrome-type compound, a cyclic hexapeptide with one alanine, two glycines, and three AHOs in which the hydroxylamino groups are acylated with malonic acid. More recently, a study on *Fusarium oxysporum* (strain FGSC 9935) reported that this fungus produces three different ferrichrome-type siderophores, ferricrocin, ferrichrome C, and malonichrome (Lopez-Berges et al., 2012). Like *F. graminearum*, *F. oxysporum* has corresponding orthologs of *NPS1* (FOXG_17422), *NPS2* (FOXG_06448), and *NPS6* (FOXG_09785).

In this study, we report characterization of strains of *F. graminearum* in which *NPS1*, *NPS2*, and *NPS6* genes are deleted in all possible combinations. Double *nps2nps6* mutant strains completely lack ability to make the intracellular siderophore ferricrocin and the extracellular fusarinine siderophores, and are augmented in the phenotypes identified in each single *nps*-deletion strain (i.e., hypersensitivity to iron depletion and oxidative stress, reduction in virulence, and abnormal sexual development), rather than the sum of each phenotype, which suggests overlap in function. We also formally demonstrate that *NPS1* is responsible for the production of the previously described second extracellular ferrichrome-type siderophore, malonichrome (Emery, 1980). Thorough characterization of the *nps1nps6* and *nps1nps2nps6* strains uncovered roles of Nps1 in stress tolerance, sexual development, and virulence to the host.

## Materials and methods

### Fungal strains and fungal and plant culture conditions

The *F. graminearum* (*G. zeae*) wild-type strain Gz3639 was used for all experiments (Table 1). Unless otherwise mentioned, all cultures were grown on complete medium [CM; (Leach et al., 1982)] at 24 C under continuous fluorescent light (Watt-Miser F34 WW/RS/WM, Warm White, General Electric). Wheat cultivar Norm was grown (four plants per #6 pot) in a greenhouse in 14 h light/10 h dark at 25 C.

**Table 1:** **Strains used in this study**.

**Strain^a^**	**Genotype**	**Designation in text**	**Designation in figures**	**Comments and/or References**
Gz3639 (NRRL 29169)	WT^b^ (*NPS1*;*NPS2*;*NPS6*)	WT	WT	WT, (Bowden and Leslie, 1999)
Gznps1-5-1	*nps1*Δ::*hygB*	*nps1*	*1*	Oide et al., 2007
Gznps2-6-1	*nps2*Δ::*hygB*	*nps2*	*2*	Oide et al., 2007
Fgnps6G-1	*nps6*Δ::*nptII*	*nps6*	*6*	Oide et al., 2006
Gznps2-6-1Δnps1-5-1	*nps1*Δ::*hygB*; *nps2*Δ::*nptII*	*nps1nps2*	*1*;*2*	Oide et al., 2007
Gznps1-5-1Δnps6-3-1	*nps1*Δ::*hygB*; *nps6*Δ::*nptII*	*nps1nps6*	*1*;*6*	This study, Gznps1-5-1 bkg^c^
Gznps1-5-1Δnps6-5-1	*nps1*Δ::*hygB*; *nps6*Δ::*nptII*	*nps1nps6*	*1*;*6*	This study, Gznps1-5-1 bkg
Gznps2-6-1Δnps6-6	*nps2*Δ::*hygB*; *nps6*Δ::*nptII*	*nps2nps6*	*2*;*6*	This study, Gznps2-6-1 bkg
Gznps2-6-1Δnps6-17	*nps2*Δ::*hygB*; *nps6*Δ::*nptII*	*nps2nps6*	*2*;*6*	This study, Gznps2-6-1 bkg
Gznps2-6-1Δnps1-5-1 Δnps6-3-2	*nps1*Δ::*hygB*; *nps2*Δ::*nptII*; *nps6*Δ::*BDS*	*nps1nps2nps6*	*1*;*2*;*6*	This study, Gznps2-6-1nps1-5-1 bkg
Gznps2-6-1Δnps1-5-1 Δnps6-11-1	*nps1*Δ::*hygB*; *nps2*Δ::*nptII*; *nps6*Δ::*BDS*	*nps1nps2nps6*	*1*;*2*;*6*	This study, Gznps2-6-1nps1-5-1 bkg

a *Nomenclature: Gz, Gibberella zeae—Fusarium graminearum; Gznps1-5-1 is single conidial isolate 1 of transformant 5 in which NPS1 was deleted from WT; Gznps2-6-1nps1-5-1 is single conidial isolate 1 of transformant 5 of Gznps2-6-1Δnps1-5-1, in which the NPS1 gene was deleted in strain Gznps2-6-1*.

b *WT carries wild-type copies of NPS1, NPS2, and NPS6*.

c *bkg, genetic background*.

### DNA manipulations and fungal transformations

Fungal genomic DNA was prepared as described previously (Oide et al., 2006, 2007). Unless otherwise mentioned, all PCR reactions were carried out with PCR master mix (Promega) following the manufacturer's recommendations. Transformation of *F. graminearum* was carried out as described previously (Oide et al., 2006, 2007). All transformants were purified by two rounds of single asexual spore isolation. All mutants used in this report are listed in Table 1.

### Construction of *F. graminearum* nps1nps6, nps2nps6 double and nps1nps2nps6 triple mutant strains

Single deletion *nps1, nps2*, and *nps6*, and double deletion *nps1nps2* mutants were constructed previously (Oide et al., 2006, 2007). Double deletion mutants *nps1nps6* and *nps2nps6* were constructed by deleting *NPS6* in the hygromycin B resistant strains Gznps1-5-1 and Gznps2-6-1 (Oide et al., 2007), respectively (Table 1). Triple *nps1nps2nps6* mutant strains were constructed by deleting *NPS6* in the hygromycin B and G418 resistant *nps1nps2* mutant strain Gznps2-6-1nps1-5-1 (Oide et al., 2007). For this, the *NPS6* ORF was partially replaced with the *Streptomyces griseochromogenes BSD* gene, which confers resistance to the antibiotic blasticidin S (BS, Invitrogen). The *BSD* gene, under the control of the *A. nidulans TrpC* promoter and terminator was PCR amplified from pBF101 (Kimura et al., 1994) with the primer pair M13F/M13R. A linear construct for transformation was prepared in the same way as described for partial deletion of *NPS6* with the gene (*bar*) for bialaphos resistance (Oide et al., 2006). Screening of transformants was carried out on complete medium without salts (CMNS) with 300 μg/ml BS. Deletion of *NPS6* was confirmed by PCR using methods described earlier (Oide et al., 2006; Inderbitzin et al., 2010).

### Stress sensitivity assays

Each strain was grown on solid minimal medium (MM) with or without the stress agent. Sensitivity to each stress was scored by measuring the colony radius of 5 day-old cultures on plates with the stress agent. Sensitivities to H_2_O_2_, the superoxide-generator, KO_2_, and the membrane-permeable iron chelator, 2-2′-dipyridyl (2DP), were examined by determining minimal inhibitory concentration (MIC) of each stress agent, as described previously (Oide et al., 2006, 2007). Briefly, a fresh stock solution of each stress agent was prepared for each experiment (1 M KO_2_ and 10 mM 2DP, in water) and the stress agents were added to MM after autoclaving (MM at approximately 48°C). Fresh MM plates with the stress agents were prepared for each experiment. All experiments were carried out in the dark. For determination of MIC of H_2_O_2_ to *F. graminearum*, MM plates with 0, 3, 6, and 12 mM H_2_O_2_ were prepared. For KO_2, *two*_ different sets of MM plates with 0, 6, 12, and 24 mM KO_2_, or with 0, 3.5, 7, 14, and 28 mM KO_2_, were prepared. For 2DP, MM plates with 0, 100, 200, and 400 μM 2DP were prepared.

To test sensitivity to iron depletion, growth of WT and different mutant strains was examined on MM and MM with 200/400 μM ferric citrate. Growing tips of mycelia were transferred from 3 day-old cultures on CM plates to fresh plates of MM with/without ferric citrate with a cork borer (3 mm diameter). The plates were incubated for 5 days under standard culture conditions. Five replicates were set up for each strain and for each condition. Average colony diameters of 5 day-old cultures were determined for each strain, and the data were analyzed by one-way analysis of variance (ANOVA) using Excel 2007.

In addition to determination of MIC of 2DP, sensitivity to 2DP of mutant strains was examined. Growing tips of mycelia were transferred by taking plugs with a cork borer (3 mm diameter) from 3 day-old cultures on CM plates to fresh plates of MM with 0, 50, and 100 μM 2DP. MM with 2DP was prepared as described above. The plates were incubated in the dark at 24°C for 4 days. Five replicates were set up for each strain and for each condition. Average colony diameters of 4 day-old cultures were determined for each strain, and the data were analyzed by ANOVA.

### Evaluation of fertility

*F. graminearum* self matings were set up on carrot juice (CJ) medium, as described previously (Oide et al., 2007). For mating, growing mycelial tips were placed in the centers of CJ agar plates, the plates were sealed with parafilm and incubated under continuous black light at 24°C for 7 days. Approximately 1 ml of sterile 2.5% Tween 60 solution was applied to the plate, and mycelia growing on the plate were knocked down with a rubber policeman, which induced sexual development. Excess Tween 60 solution was discarded and the unsealed plates were incubated under the original conditions for an additional 7 days (Oide et al., 2007).

Fertility was examined based on the number of perithecia per plate and the number of asci per perithecium. At least, five replicates were set up for each self and the number of perithecia per plate was recorded for each plate. At least 20 perithecia were opened for each self and the number of asci was recorded for each perithecium. All data were analyzed by ANOVA.

Fertility of *F. graminearum* selfs supplied with iron was examined in the same way as described above and previously (Oide et al., 2007). Briefly, a fresh stock solution of ferric citrate (10 mM in water) was applied to carrot juice medium before autoclaving, so that the final concentration was 100, 125, 200 or 250 μM.

### Evaluation of virulence

Virulence assays of *F. graminearum* were carried out as described previously (Oide et al., 2006). At least five independent spikes were inoculated per strain and per assay. In point-inoculation of wheat spikes, local infection, as well as systemic infection, was evaluated. The time required for completion of local infection after inoculation was recorded for each spike. When an inoculated spikelet became completely bleached, local infection was considered finished. At least five spike replicates were set up for each strain, and the average time for completion of local infection was determined for each strain. The data were statistically analyzed by ANOVA. Experiments were repeated at least three times.

### Isolation, identification, and semi-quantitative analysis of *F. graminearum* siderophores

Strains were pregrown under iron replete conditions in Fusarium MM made according to the Fusarium Laboratory Manual (Leslie and Summerell, 2006), containing the standard amount of iron (100 ml flasks, 22°C, 150 rpm). Mycelia from dense 4 day-old cultures were harvested by vacuum filtration using sterile Buchner funnels fitted with sterile filter paper. The mycelia were washed with about 25 ml of iron-free MM, then scraped off the filter paper and transferred to empty Petri dishes. After determination of the wet weight the mycelia were transferred into fresh Fusarium MM without iron. Initially, in all cases, a volume of 6 ml/g wet weight was added during the medium replacement, resulting in cultures with a volume of about 50 ml (±10%). The resuspended cultures were incubated in this iron starvation medium on a rotary shaker at 150 rpm at 22°C for 3, 6, 11, and 15 days. At harvest, the cultures were first filtered through glass wool to remove the mycelium then clarified by centrifugation to remove any remaining mycelium, including conidiospores formed during cultivation in iron-free medium. FeCl_3_ was added to the cleared supernatant (10 mM final), which was centrifuged for 10 min at 12,000 rpm. The supernatants were analyzed with LC-UV-MS. HPLC grade methanol was purchased from J. T. Baker (Deventer, The Netherlands) and formic acid (analytical grade) was obtained from Sigma-Aldrich (Vienna, Austria). Water was purified successively by reverse osmosis and a Milli-Q plus system from Millipore (Molsheim, France). Pure ferricrocin, as well as a mixture containing fusigen, dimerum acid, coprogen, neocoprogens I and II and triacetylfusarinine C (TAFC) were purchased from EMC Microcollections (Tübingen, Germany) and used as qualitative standards.

HPLC separation was carried out on a Gemini-C18 column (4.6 × 150 mm, 5 μm) equipped with a 4 × 3 mm security guard cartridge (both from Phenomenex, Torrance, CA) on an 1100 Series HPLC system (Agilent, Waldbronn, Germany). Eluent A consisted of 5% aqueous methanol with 1% formic acid, while eluent B was 99% methanol with 1% formic acid. After a hold time of 1.5 min at 100% A, the proportion of B was increased linearly to 100% within 10 min, followed by a hold time of 2.5 min at 100% B and 6 min column re-equilibration at 100% A. Total runtime was 20 min at a flow rate of 750 μl/min. The column was kept at 25°C and an injection volume of 25 μl was used. The diode array detector was set to a wavelength of 435 nm. After the UV detector, the column effluent was transferred via a six-port valve either to the mass spectrometer (between 4 and 14 min) or to the waste. Full scan mass spectra (using Q1) were recorded from m/z 500 to 1200 on a QTrap LC-MS/MS system (Applied Biosystems, Foster City, CA) equipped with a TurboIonSpray ESI source in positive ion mode using Analyst version 1.4.1. The following settings were used for the MS: source temperature 400°C, curtain gas 25 psi, gas 1 (sheath gas) 30 psi, gas 2 (drying gas) 75 psi, ion spray voltage 5000 V, declustering potential 30 V, scan time 1 s.

High resolution mass spectrometric measurements were performed on a 6550 iFunnel Q-TOF mass spectrometer (Agilent Technologies, Waldbronn, Germany) operated in MS and MS/MS mode. Chromatographic separation was achieved using a Gemini C6-Phenyl C-6 column (2.0 × 50 mm, 3 μm, Phenomenex) using a linear gradient from 5 to 100% methanol, containing 40 mM formic acid and 20 mM ammonium formate.

## Results

### Metabolite analysis

Three and a half decades ago, Emery described the siderophore, malonichrome, produced by a *F. roseum* strain (ATCC12822) (Emery, 1980). While *F. roseum* is generally considered as a synonym of *F. graminearum*, this strain is, in fact, most closely related to *F. equiseti* based on the nucleotide sequence available for the LSUrDNA D1D2 from the equivalent strain NBRC 8502 (Culture Collection Division of the Biological Resource Center, Japan). The established structure for malonichrome is that of a ferrichrome-type compound, specifically, a cyclic hexapeptide with one alanine, two glycines and three AHOs, in which the hydroxylamino groups are acylated with malonic acid (Emery, 1980).

**Figure 1:**
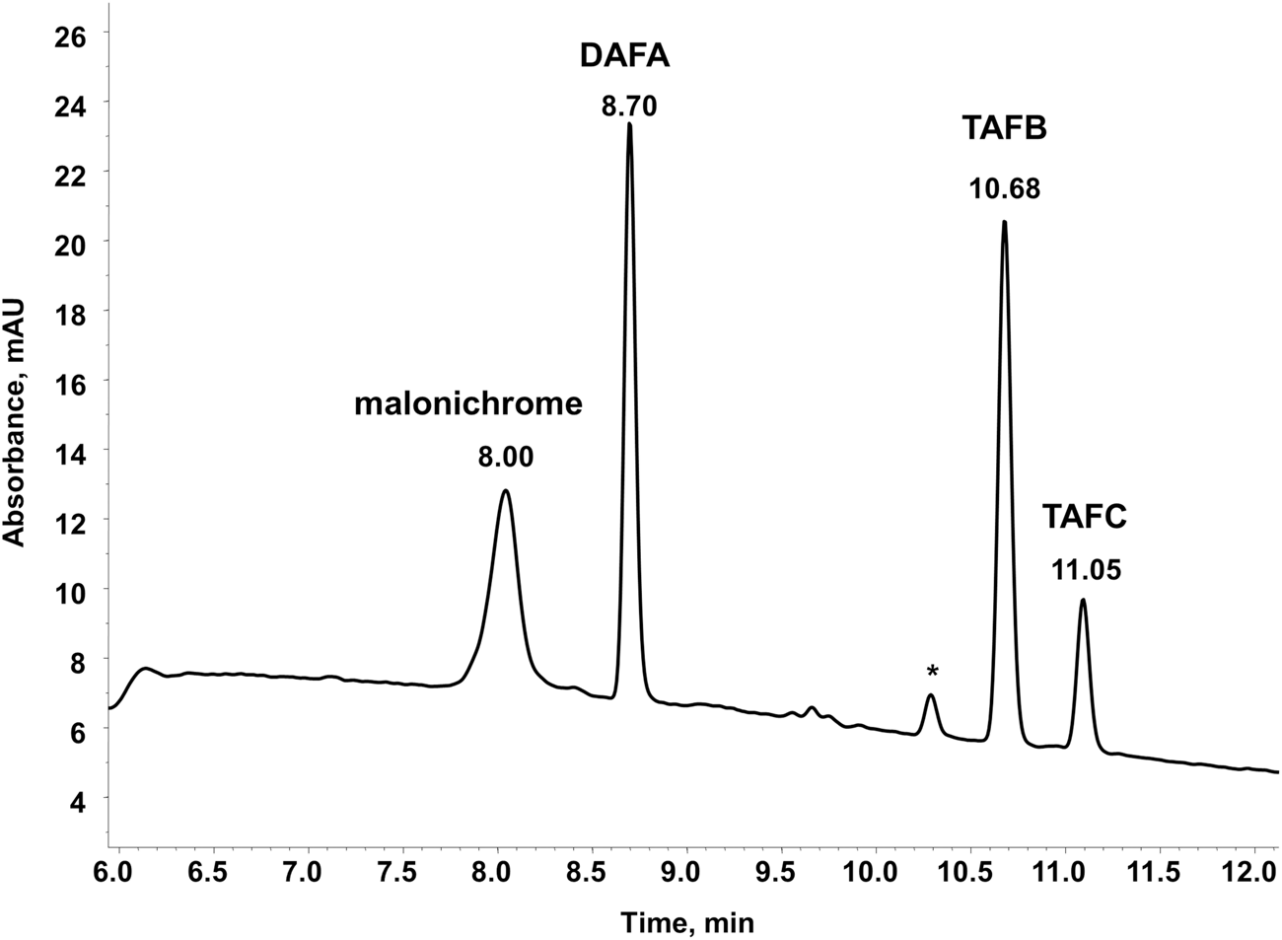
**LC-UV chromatogram of wild-type *F. graminearum***. The fungus was cultivated in liquid MM without iron for 6 days. FeCl_3_ was added before measurement of the supernatant at 435 nm. Siderophores present are DAFA, malonichrome, TAFB, and TAFC. ^*^ denotes a small interfering peak, also visible in pure solvent.

**Figure 2:**
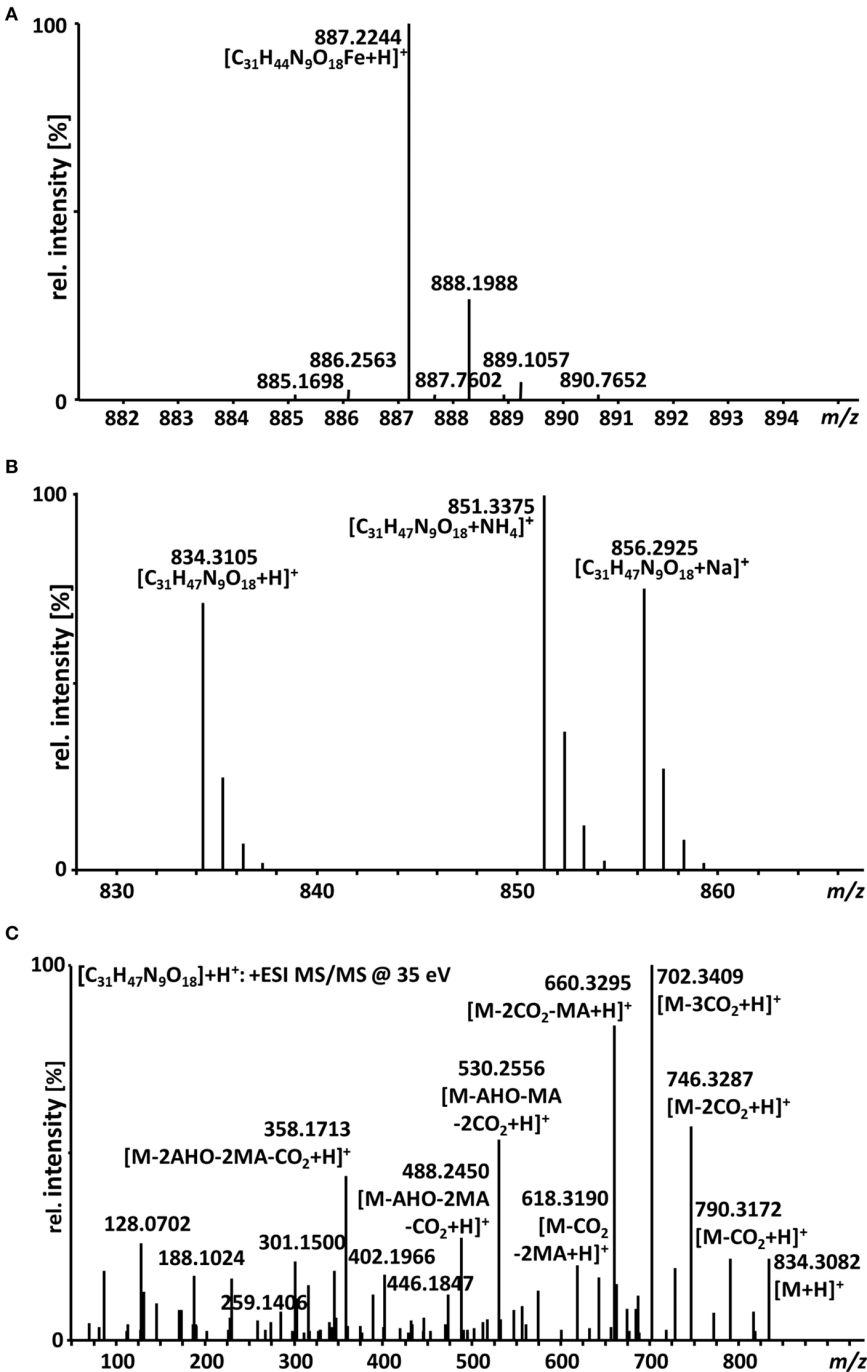
**LC-HR-MS spectra of malonichrome**. **(A)** Isotope pattern of the [M+H]^+^ ion cluster of malonichrome (ferri-form). **(B)** LC-HR-MS spectrum of malonichrome (desferri-form), showing [M+H]^+^, [M+NH_4_]^+^ and [M+Na]^+^ ions. **(C)** LC-HR-MS/MS spectrum of malonichrome (desferri-form) at a collision energy of 35 eV.

**Figure 3:**
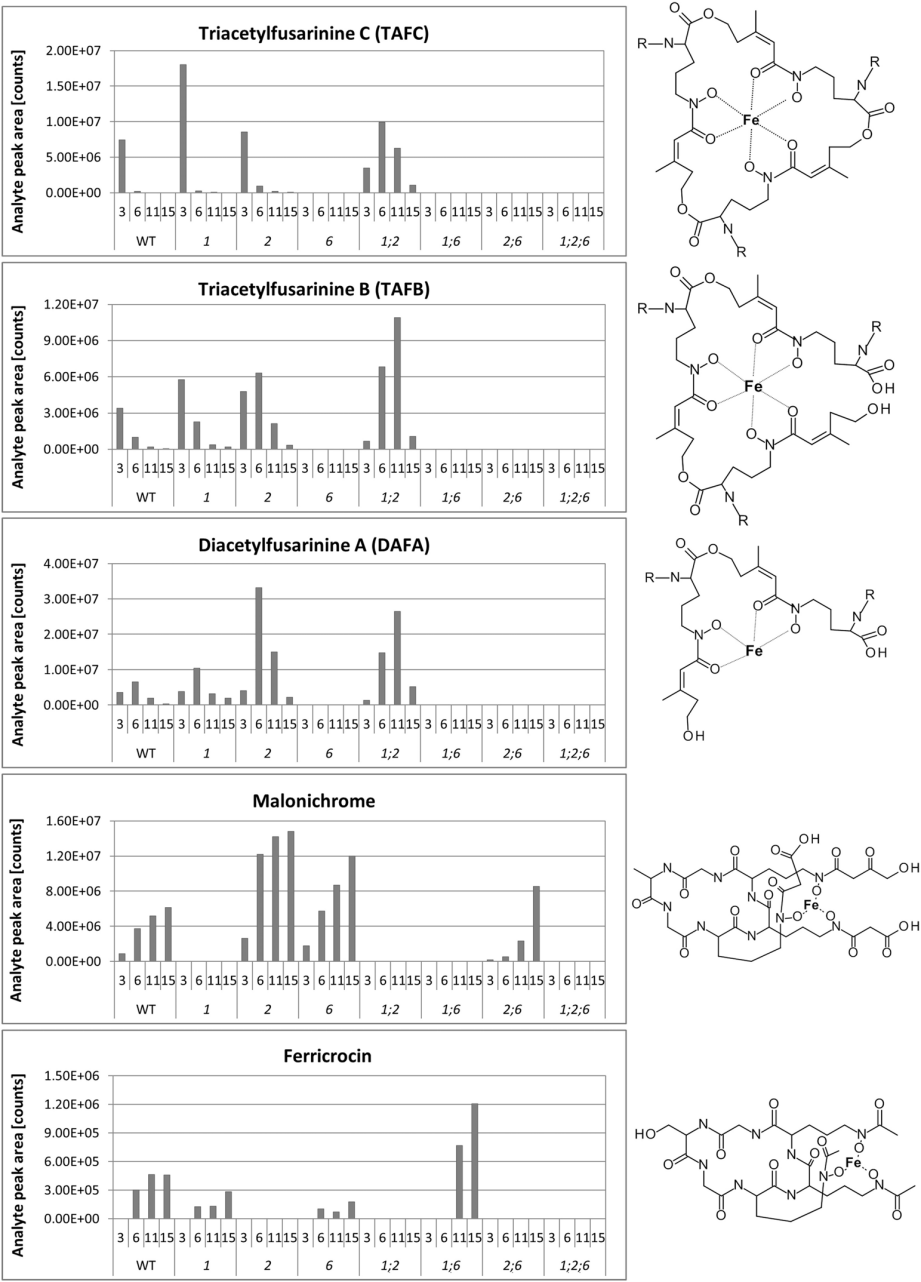
**Siderophores produced by wild-type and mutant strains**. Siderophores produced by WT Gz3639 and mutant strains (see Table 1) cultivated in MM and subsequently starved in iron-free MM as described in Materials and Methods. Samples were taken 3, 6, 11, and 15 days after shift to iron-starvation medium. Supernatants were analyzed for siderophores by HPLC as described in the Materials and Methods. X-axis: upper row, days after shift to iron-free medium, lower row, strains investigated. Y-axis: relative concentrations (arbitrary units), R (TAFC, TAFB, DAFA): acetyl. Note, metabolites are missing in all strains with deletions of corresponding genes, as expected. Malonichrome is present in strains with a WT *NPS1* gene and absent from strains deleted for this gene.

A big advantage of iron-chelating compounds is their UV absorbance at 435 nm, which renders analytical detection straightforward. A LC-UV chromatogram of the culture supernatant of WT *F. graminearum*, grown under iron starvation for 6 days, is provided as Figure 1. Concurrent MS analyses of the peak at 8.00 min identified compounds with the mass and sum formula corresponding to malonichrome (after iron supplementation) and desferri-malonichrome (in the absence of iron) (Figures 2A,B). The enhanced resolution scan of the compound with the mass of malonichrome shows clearly that this compound is able to bind iron. The signal with *m/z* 885 originates from binding of ^54^Fe (making up 6% in naturally occurring isotope mixtures) whereas the main peak with *m/z* 887 is derived from ^56^Fe (92%) and the peak with *m/z* 888 from ^57^Fe (2%). LC-HR-MS measurements yielded the highest intensity at *m/z* 887.2244 for the [M+H]^+^ ion of the ferri-form. This confirms the sum formula of C_31_H_44_FeN_9_O_18_ with a deviation from the theoretical mass of +1.4 ppm (Figure 2A). For the desferri-form *m/z* values of 834.3105 (Δ+0.9 ppm), 851.3375 (Δ+0.2 ppm) and 856.2925 (Δ +0.7 ppm) have been found, which match the protonated ion, the ammonium adduct and the sodium adduct of C_31_H_47_N_9_O_18_, respectively (Figure 2B). Also the collision induced dissociation pattern in LC-MS/MS experiments (Figure 2C) is consistent with the structure proposed by Emery (1980). Cleavage of carbon dioxide from the three malonic acid ester (MA) groups, cleavage of the respective MA groups as well as cleavage of one or both AHO (including MA) groups from the molecule can be explained from the MS/MS spectrum. We therefore conclude that the compound identified in *F. graminearum* is malonichrome. As evident from Figure 3, malonichrome is missing in samples of all mutants lacking *NPS1* (*nps1*, *nps1nps2*, *nps1nps6* and the triple mutant *nps1nps2nps6*), demonstrating, formally, that the *NPS1* gene is required for malonichrome biosynthesis.

In agreement with our previous publication (Oide et al., 2007), ferricrocin was missing in samples of all mutants carrying a deletion of *NPS2* (Figure 3). Unexpectedly, however, we detected ferricrocin in the culture supernatant of WT, at later time points (after 11 days of iron starvation; not visible in Figure 1), indicating that this “intracellular siderophore” is not as strictly intracellular as previously thought. Furthermore, the supernatant of the *nps1nps6* mutant which is expected to produce only ferricrocin, had an approximately 2X increase in ferricrocin accumulation compared to WT (Figure 3). Figure 3 also shows early accumulation of TAFC and transient formation of its derivatives triacetyfusarinine B (TAFB) and diacetylfusarinine A (DAFA) with hydrolyzed ester bonds. As noted by Emery for *F. roseum* (Emery, 1980), we observed a switch from production of the ester type fusarinines to the peptide type siderophore malonichrome as the major extracellular siderophore for WT *F. graminearum* after extended culture. Interestingly, production of TAFC and the hydrolysis product TAFB was maintained longer in the mutant lacking the other two siderophores (*nps1nps2*).

### Growth and sensitivity to ROS and iron depletion

Single, double, and triple mutant strains (Table 1) were used to perform phenotypic tests. Single gene deletions of either *NPS1* or *NPS2* do not affect tolerance to oxidative stress mediated by H_2_O_2_ and KO_2_ as described previously (Oide et al., 2007). Likewise, *nps1nps2* strains show WT tolerance. Deletion of *NPS6*, however, compromises tolerance to both types of ROS (Oide et al., 2006). *nps1nps6* strains showed *nps6*-like sensitivity to ROS, while double deletion of *NPS2* and *NPS6* further increased sensitivity to H_2_O_2_ and KO_2_ compared to *nps6* strains (Table 2). Triple *nps1nps2nps6* mutant strains were indistinguishable from double *nps2nps6* mutant strains in terms of sensitivity to H_2_O_2_ (see Table 2 for MICs). Similarly, there was no difference in MIC of KO_2_ to *nps2nps6* and *nps1nps2nps6* strains, when sensitivity to 3, 6, 12, and 24 mM KO_2_ was examined. A subtle reduction in growth, however, was observed for the latter strain on MM with 3 mM KO_2_ compared to the former (data not shown). When examined on MM with 3.5, 7, 14, and 28 mM KO_2_, MIC of KO_2_ to *nps1nps2nps6* strains was 3.5 mM, while that of *nps2nps6* strains was 7 mM (Table 2), indicating that deletion of *NPS1* enhances the sensitivity of *nps2nps6* strains to KO_2_. These observations allow the conclusion that *NPS1* and *NPS2* contribute to oxidative stress tolerance.

**Table 2:** **MIC of H_2_O_2_, KO_2_, and 2DP to WT and mutant strains**.

**Strain**	**Stress**		
	**H_2_O_2_ (mM)**	**KO_2_ (mM)^b^**	**2DP (μM)**
WT	12 > ^a^	24/28	400
*nps6*	6	12/7	200
*nps2nps6*	3	6/7	200
*nps1nps6*	6	12/7	200
*nps1nps2nps6*	3	6/3.5	200

a *“>” indicates that growth of a strain was observed on MM with the maximal concentration of a stress agent tested in this study. Thus, MIC of the stress agent to that strain is higher than the maximal concentration tested (e.g., MIC of H_2_O_2_ to WT is higher than 12 mM)*.

b *MIC of KO_2_ to F. graminearum strains was examined in two different scales. In the first series of experiments (left of forward slash), sensitivity to 3, 6, 12, and 24 mM KO_2_ was examined. In the second series, sensitivity to 3.5, 7, 14, and 28 mM KO_2_ was examined*.

**Figure 4:**
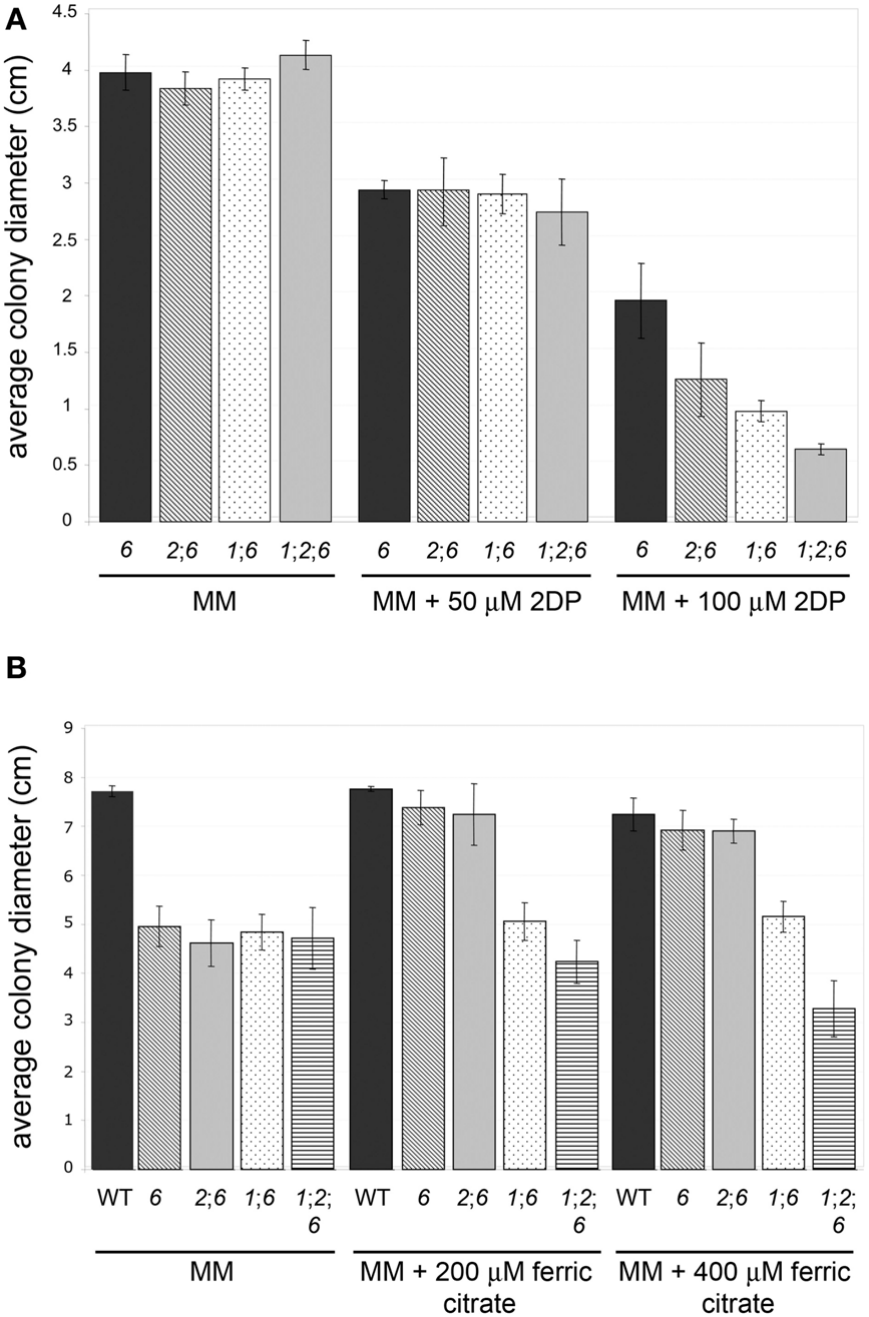
**Sensitivity to iron depletion of WT and mutant strains**. **(A)** *nps2nps6*, *nps1nps6*, and *nps1nps2nps6* strains show further increased sensitivity to 2DP, compared to the *nps6* strain. Average colony diameters of 4 day-old cultures on MM with/without 2DP are shown. Strains used are as in **(B)**. Error bars indicate 95% confidence intervals. A statistically significant reduction in growth on MM with 100 μM 2DP was observed for the *nps2nps6* and *nps1nps6* strains, compared to the *nps6* strain. The *nps1nps2nps6* strains show further reduced growth on MM with 100 μM 2DP, compared to the *nps2nps6* or *nps1nps6* strains. No significant difference in growth was observed on MM among all strains. **(B)** Application of iron enhances growth of *nps6* and *nps2nps6*, but not *nps1nps6* and *nps1nps2nps6* strains. Average colony diameters of 5 day-old cultures of WT (strain Gz3639), *nps6* (strain Fgnps6G-1), *nps2nps6* (Gznps2-6-1nps6-17), *nps1nps6* (Gznps1-5-1nps6-3-1), and *nps1nps2nps6* (Gznps2-6-1nps1-5-1nps6-3-2) strains on MM with/without iron are shown. Error bars indicate 95% confidence intervals. A statistically significant reduction in growth on MM was observed for all mutant strains compared to WT. Application of 200 or 400 μM ferric citrate restored WT growth to the *nps6* and *nps2nps6* strains, indicating that the growth defect of these strains on MM is due to iron deficiency. In contrast, application of iron did not enhance growth of the *nps1nps6* strains. Application of 400 μM ferric citrate reduced growth of the *nps1nps2nps6* strains, implying that they are hypersensitive to iron overload.

*nps1*, *nps2*, as well as *nps1nps2* strains show the same tolerance to iron depletion as the WT strain, whereas *nps6* strains are compromised in growth under low iron conditions. Judged by MICs of 2DP, no difference in sensitivity to iron depletion was observed among *nps6*, *nps1nps6*, *nps2nps6*, and *nps1nps2nps6* strains (Table 2). Nevertheless, a reduction in growth on MM with 100 μM 2DP was observed for the latter three compared to *nps6* strains (Figure 4A), and growth of *nps1nps2nps6* strains was most severely affected. As previously published (Oide et al., 2006), *nps6* strains have a growth defect on MM due to starvation for iron, and exogenous application of iron restores WT growth to *nps6* strains (Figure 4B). When cultured on MM, growth of *nps1nps6*, *nps2nps6*, and *nps1nps2nps6* strains was reduced to the same extent as observed for *nps6* strains (Figure 4B). Iron-dependent restoration of growth was observed for *nps2nps6* strains but not for *nps1nps6* strains. Moreover, increasing concentrations of iron further attenuated growth of *nps1nps2nps6* strains, suggesting that the strains are hypersensitive to iron overload. Overall, these results indicate that *NPS1* is involved in iron metabolism of *F. graminearum*.

### Sexual reproduction

Earlier we reported that *nps2* strains of self-compatible *F. graminearum* (*G. zeae*) are defective in ascus/ascospore development, although they show WT perithecium development. In contrast, *nps1* and *nps6* deletion strains are not affected in sexual development. *nps1nps2* strains are like *nps2* strains in terms of fertility (Oide et al., 2007).

**Figure 5:**
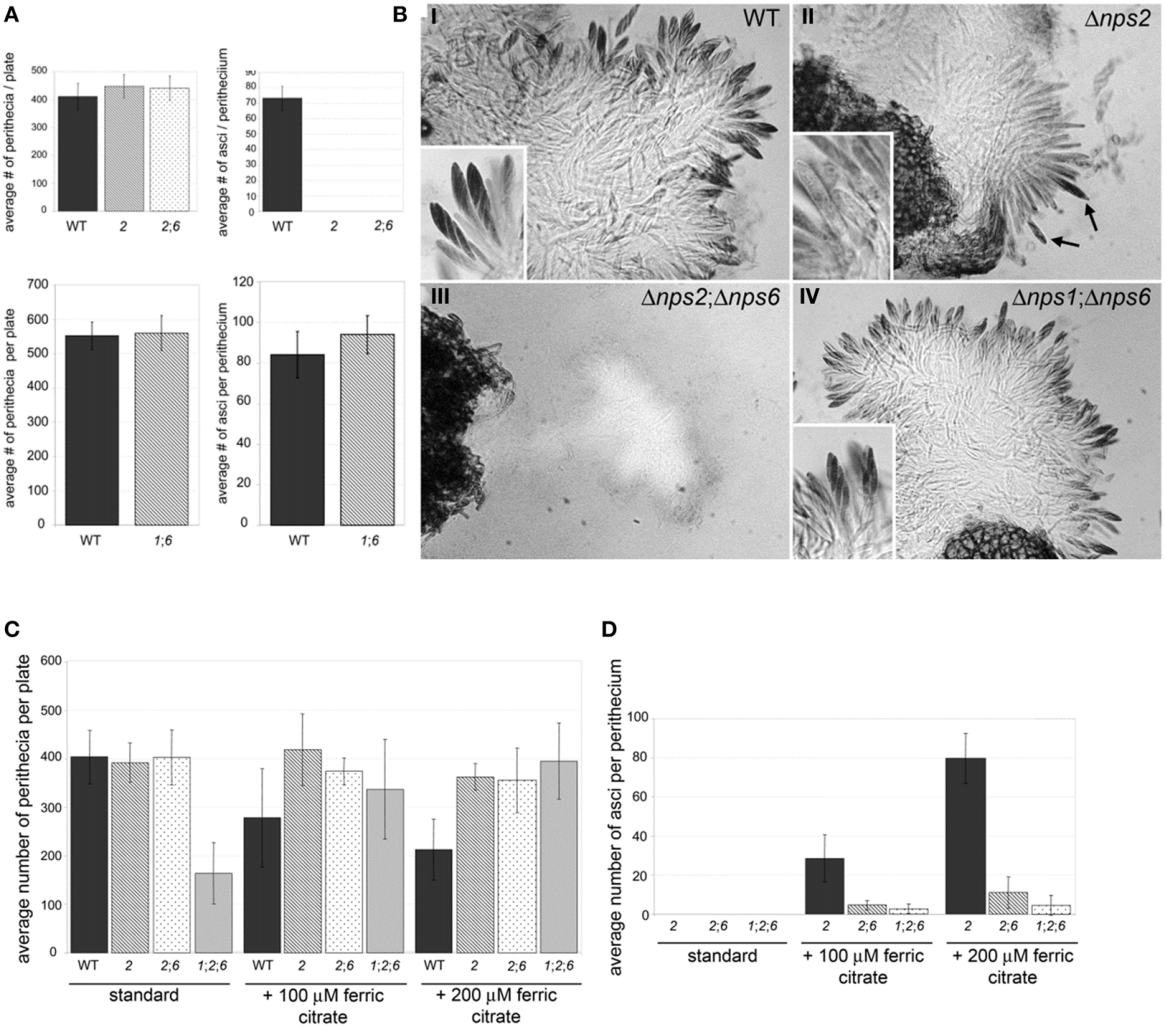
**Nps2 is required for sexual reproduction of self-compatible strains but Nps6 plays a role when Nps2 is absent**. **(A)** *nps2nps6* and *nps1nps6* strains form perithecia as well as WT strains do. Top and bottom, left, average number of perithecia per plate is shown for WT, *nps2* (*2*), *nps2nps6* (*26*), and *nps1nps6* (*16*) selfs. Ten replicates were set up for each strain. Error bars indicate 95% confidence intervals. No significant difference was observed in the number of perithecia per plate between WT, and any of the mutant strains. Top, right, *nps2nps6* strains fail to develop asci and ascospores. WT, *nps2*, and *nps2nps6* selfs are shown. As observed in *nps2* strains, *nps2nps6* strains fail to develop asci and ascospores. Bottom, right, *nps1nps6* strains are as fertile as WT. Average number of asci per perithecium is shown for WT and *nps1nps6* selfs. Thirty perithecia were opened for each self. Error bars indicate 95% confidence intervals. No significant difference was observed in the number of asci per perithecium between WT and *nps1nps6* selfs. **(B)** *nps2* strains which develop immature ascus-like structures (II, arrows), however no ascus-like structures were found in perithecia developed in the *nps2nps6* selfs (III, arrows), indicating that sexual development of the *nps2nps6* strain is more severely affected than that of the *nps2* strain. *nps1nps6* strains are as fertile as WT (compare I and IV). Magnification is × 200, inserts × 500. **(C)** *nps1nps2nps6* strains are more affected in sexual development than *nps2nps6* strains. Application of iron restores ability to develop asci and ascospores to *nps2*, *nps2nps6*, and *nps1nps2nps6* strains. Five replicates were set up for each self and for each condition. A statistically significant reduction in the number of perithecia per plate was observed in the *nps1nps2nps6* selfs, compared to WT, *nps2*, and *nps2nps6* selfs. Exogenous application of iron enhanced perithecium development of the *nps1nps2nps6* selfs, indicating that reduced perithecium development is due to iron deficiency. When iron is applied, a reduction in perithecium development was observed in WT selfs. **(D)** Twenty perithecia were opened for each self and for each condition. Error bars indicate 95% confidence intervals. Although no statistically significant difference was observed in the number of asci per perithecium between the *nps2nps6* and *nps1nps2nps6* selfs, the number tended to be smaller in the *nps1nps2nps6* compared to the *nps2nps6* selfs. A statistically significant difference in the number of asci per perithecium was observed between the *nps2* and *nps2nps6* strains, further demonstrating that *nps2nps6* strains are more affected in sexual development.

When the *nps1* deletion was combined with the *nps6* deletion, *nps1nps6* strains were as fertile as WT strains, indicating that *NPS1* and *NPS6* are dispensable for sexual development in the presence of *NPS2* (Figures 5A,B). Double *nps2nps6* mutants formed perithecia that were WT in number and in morphology when selfed (Figure 5A top, left) but, like *nps2* strains, showed defects in ascus development (Figure 5A top, right). However, in contrast to perithecia developed by *nps2* strains, in which immature ascus-like structures are often observed (note Figure 5B, top right, arrows), perithecia developed by *nps2nps6* strains were devoid of any ascus-like structures (Figure 5B bottom, left). Application of iron restored ability to develop asci and ascospores to both types of strain, but there were fewer asci per perithecium in the *nps2nps6* compared to the *nps2* selfs (Figures 5C,D). Taken together, these results indicate that iron metabolism during sexual development is impaired more severely in *nps2nps6*, compared to *nps2* strains.

Although single *nps1* or double *nps1nps6* strains did not show any defect in sexual reproduction, triple deletion *nps1nps2nps6* strains showed a reduction in the number of perithecia developed in selfs, compared to the WT, *nps2*, or *nps2nps6* selfs (Figure 5C; Table 3). As observed for the *nps2nps6* selfs, the perithecia developed in the *nps1nps2nps6* selfs had WT morphology but lacked any ascus-like structures (not shown). When iron was applied exogenously, the *nps1nps2nps6* selfs formed as many perithecia as the *nps2* or *nps2nps6* selfs (Figure 5C), indicating that reduced perithecium development of the *nps1nps2nps6* selfs is attributable to iron deficiency. Application of iron also restored ability to form asci and ascospores to *nps1nps2nps6* strains (Figure 3D), but the number of asci/perithecium of the *nps1nps2nps6* selfs was less than that of the *nps2* selfs (Figure 3D). No statistically significant difference between the *nps2nps6* and *nps1nps2nps6* selfs was observed in the number of asci/perithecium, although the number of asci tended to be less in the latter compared to the former. The observations indicate that *NPS1* plays a role in maintaining iron homeostasis during sexual development.

**Table 3:** **Selfs set up in this study**.

**Self**	**Strains^a^**
WT	Gz 3639
*nps2*	Gznps2-6-1
*nps1nps6*	Gznps1-5-1nps6-3-1
	Gznps1-5-1nps6-5-1
*nps2nps6*	Gznps2-6-1nps6-6
	Gznps2-6-1nps6-17
*nps1nps2nps6*	Gznps2-6-1nps1-5-1nps6-3-2
	Gznps2-6-1nps1-5-1nps6-11-1

a *For strains, see Table 1*.

### Virulence

**Figure 6:**
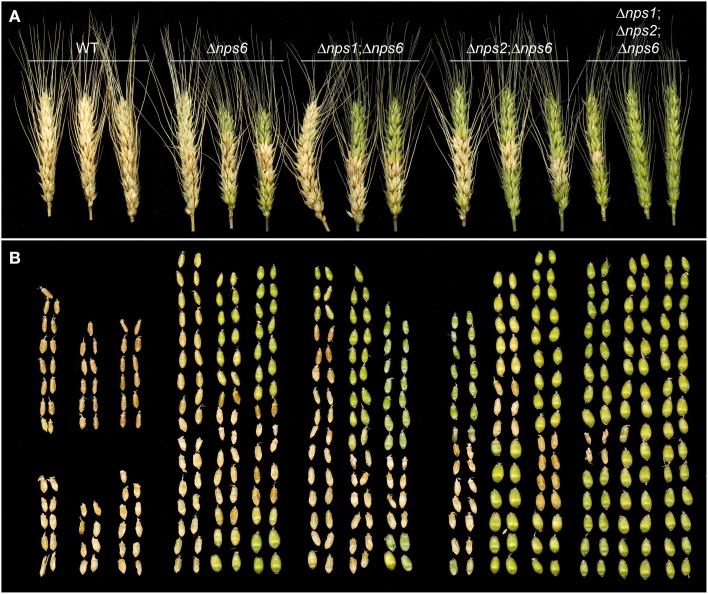
**Virulence of WT, *nps6*, *nps1nps6*, *nps2nps6*, and *nps1nps2nps6* strains to wheat**. **(A)** Wheat spikes challenged by WT (strain Gz3639), *nps6* (strain Fgnps6G-1), *nps1nps6* (strain Gznps1-5-1nps6-3-1), *nps2nps6* (strain Gznps2-6-1nps6-17), and *nps1nps2nps6* (strain Gznps2-6-1nps1-5-1nps6-3-2), strains, 16 days after inoculation. Spikes inoculated with the *nps2nps6* strain (second set from right) are less damaged than those inoculated with the *nps6* strain (second set from left). Spikes inoculated with the *nps1nps6* strain (middle set) were as severely damaged as those inoculated with *nps6* strains. Spikes inoculated with the *nps1nps2nps6* strain (right set) show further reduced virulence compared to the *nps2nps6* strain and were almost intact. **(B)** Kernels taken from the spikes shown in **(A)**. Relative positions of kernels correspond to those of the spikes shown in **(A)**. Generally, spikes challenged by *nps2nps6* strains contained more healthy, green kernels (note tops of spikes) than did those challenged by *nps6* strains, demonstrating that the *nps2nps6* strain is more reduced in virulence for systemic infection of wheat spikes, than the *nps6* strain is. Kernels in the spikes infected by the *nps1nps6* strains were as severely damaged as those in the spikes infected by the *nps6* strain. Most of the kernels in the spikes infected by the *nps1nps2nps6* strains appeared undamaged. Overall, these data demonstrate that the *nps1nps6* strain is as virulent to wheat spikes as the *nps6* strain is, and that the *nps1nps2nps6* strain is further attenuated in virulence to wheat spikes, compared to the *nps2nps6* strain.

**Figure 7:**
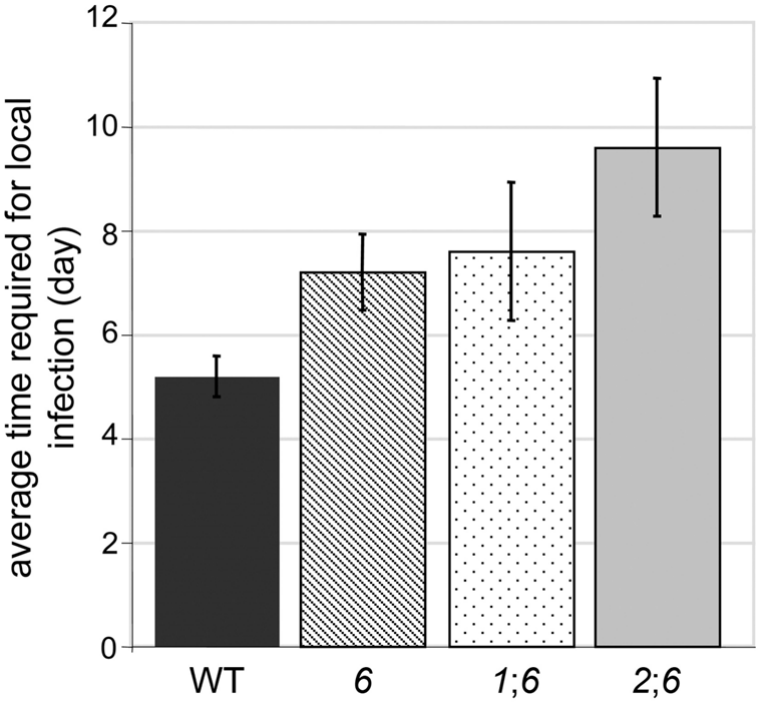
**Average time required for completion of local infection of wheat spikelets by WT, *nps6* (*6*), *nps1nps6* (*16*), and *nps2nps6* (*26*) strains**. Note that the *nps1nps2nps6* strain was not included, as most of the spikelets inoculated with the *nps1nps2nps6* strains were asymptomatic until the end of infection assays (i.e., 16 days after inoculation). No significant difference was observed in the time for local infection between the *nps6* and *nps1nps6* strains, indicating that the *nps1nps6* strain is as virulent for local infection of wheat spikes as the *nps6* strain is. Generally, it takes longer for the *nps2nps6* strain to complete local infection, compared to the *nps6* strain.

Deletion of *NPS6* impairs virulence of *F. graminearum* to wheat (Oide et al., 2006), whereas *nps1*, *nps2*, *nps1nps2* strains retain WT virulence to the host (data not shown). A subtle reduction in virulence was observed for *nps2nps6* strains, compared to *nps6* strains (Figure 6, compare second from left wheat head sets with second from right sets). Generally, *nps2nps6* strains took longer to complete local infection than did *nps6* strains, although variation was observed among individual infection events (Figure 7). Reduced virulence of *nps2nps6* strains was also observed in systemic infection. In general, the spikes challenged by *nps2nps6* strains were less damaged than those challenged by *nps6* strains (Figure 6A). Similarly, kernels in spikes infected by *nps2nps6* strains (Figure 6B, second from right) were less damaged compared to those in spikes infected by *nps6* (Figure 6B, second from left) or WT strains (Figure 6B, left). Again, some variation in severity of symptom was observed among individual spikes challenged by the *nps6* and *nps2nps6* strains. Like *nps6* strains, *nps1nps6* strains showed reduced virulence to wheat (Figures 6A,B, 7), however, no significant difference was observed between these strains in either local or systemic infection of wheat.

The triple mutant *nps1nps2nps6* strain almost completely lost virulence to wheat. In point-inoculation assays of spikes, *nps1nps2nps6* strains developed moderate symptoms only occasionally (Figures 6A,B, right image sets). Generally, about 80% of the spikes inoculated with the *nps1nps2nps6* strain were asymptomatic, even though the spikes were incubated up to 16 days post-inoculation. These data demonstrate that simultaneous deletion of *NPS1*, *NPS2* and *NPS6* in *F. graminearum* leads to further attenuated virulence on wheat, compared to *nps2nps6* strains.

## Discussion

### Malonichrome production requires NPS1

We demonstrate here that, in addition to the ferricrocin (intracellular) and TAFC (extracellular) trihydroxamate siderophores biosynthesized by the Nps2 and Nps6 proteins, *F. graminearum* produces a second extracellular siderophore, malonichrome, described in 1980 as an extracellular siderophore of *F. equiseti (F. roseum*). Biosynthesis of this siderophore did not occur in mutants with disruptions of the *NPS1* gene (Figure 3), thus the function of Nps1 can definitively be assigned. Previously, we proposed that the *F. graminearum* Nps1 protein may be partially functional and able to produce dihydroxamates, but not trihydroxamates (Oide et al., 2007). This hypothesis was falsified by our current experiments.

### Malonichrome plays a role in iron homeostasis

Phenotypic characterization of *nps1nps6* and *nps1nps2nps6* strains implicates malonichrome in iron management in *F. graminearum*. Although no difference was observed in terms of MIC of 2DP, a reduction in growth was observed for *nps1nps6* strains compared to *nps6* strains in the presence of 100 μM 2DP. When cultured on MM, growth of *nps6* and *nps1nps6* strains was reduced to a similar extent compared to WT. In contrast to *nps6* strains, where WT growth is restored by iron (Oide et al., 2006), growth of *nps1nps6* strains was unaffected by exogenous application of iron, suggesting that malonichrome contributes to iron acquisition of *F. graminearum* in the absence of *NPS6*. This idea is further supported by the observation of *nps1nps2nps6* strains, which showed a subtle but reproducible increase in sensitivity to 2DP compared to *nps2nps6* strains. A sexual development phenotype unique to *nps1nps2nps6* strains was also observed. In selfs of these strains, a reduction in the number of perithecia was observed, in addition to complete sterility in terms of ascus/ascospore development. The reduction in numbers of perithecia was not observed in any other mutant strain, including *nps2*, *nps1nps2* (Oide et al., 2007), and *nps2nps6* strains. Application of iron facilitated perithecium formation of *nps1nps2nps6* strains, indicating that starvation for iron accounts for the developmental defect. Overall, our findings provide evidence that malonichrome produced by Nps1 is involved in iron metabolism during reproductive as well as vegetative development of *F. graminearum*.

### Extracellular siderophores produced by *NPS6* are of primary importance for iron acquisition

Here we show that the primary extracellular siderophore, which depends on an intact *NPS6* gene, is TAFC, a cyclic peptide consisting of three *N*^5^-cis-anhydromevalonyl-*N*^5^-hydroxy-*N*^2^-acetyl-l-ornithine residues linked by ester bonds. TAFC binds iron with very high affinity. In order to be able to transfer the tightly bound iron to other carriers (including the intracellular siderophore), the molecule has to be successively destroyed by hydrolysis of the ester bonds (Haas, 2014). We detected the linearized derivative (TAFB) generated by opening of one ester bond in TAFC, and the cleavage product (DAFA) generated by opening two ester bonds and loss of one *N*^5^-cis-anhydromevalonyl-*N*^5^-hydroxy-*N*^2^-acetyl-l-ornithine unit (see Figure 3). In agreement with the proposed product/precursor relationship, all of these metabolites depended on the presence of the *NPS6* gene (Figure 3). With increasing incubation time under iron depletion conditions, TAFC and its hydrolysis products were replaced by malonichrome in WT and even more pronounced in *nps2* strains. The biological significance of this shift remains a matter of future research. As already pointed out by Emery (1980), malonichrome is less efficient in mediating iron uptake than TAFC in *F. roseum*/*equiseti*. Given that the impact of *nps1* deletion is apparent only in the absence of *NPS6*, this seems to be the case also for *F. graminearum*.

### Ferricrocin an extracellular siderophore?

The ferrichrome type siderophore, ferricrocin is generally considered to be an intracellular siderophore. Yet, we observed ferricrocin in culture filtrates of WT as well as *nps1nps6* mutants that still carried the WT *NPS2* gene. Thus, designation of ferricrocin as strictly intracellular appears to be an oversimplification. Although ferricrocin is generally recognized as an intracellular siderophore in *A. fumigatus*, trace amounts can be detected in culture supernatants (Schrettl et al., 2007). The mutualistic grass endophyte *Epichloë festucae sidN* gene encodes a siderophore synthetase producing a novel extracellular ferrichrome-type siderophore, epichloënin A (Koulman et al., 2012). Also for *S. pombe*, which contains only one siderophore biosynthesis gene in its genome (Bushley et al., 2008), the resulting hydroxamate-type siderophore, ferrichrome, is accumulated both intracellularly and excreted (Schrettl et al., 2004).

An obvious question is if the *F. graminearum* secreted ferricrocin can act as an extracellular siderophore or not. Exogenous application of ferricrocin restores the WT fertility to *nps2* strains (Oide et al., 2007) indicating that *F. graminearum* can take up ferricrocin from extracellular spaces. However, our observation on *nps1nps6* strains casts doubt on the contribution of extracellular ferricrocin to iron uptake. Although this mutant accumulates increased amounts of extracellular ferricrocin compared to other strains, iron supplied exogenously failed to mitigate the growth defect of the *nps1nps6* mutant on MM. Iron needs to be released from siderophores after entering fungal cells. Due to its property as an iron storage molecule, ferricrocin iron release may be strictly regulated, and therefore iron bound to ferricrocin may not be a good source of nutritional iron.

Overall, these findings confirm the previous conclusion that TAFC and its derivatives, the primary extracellular siderophores, play a pivotal role in iron acquisition and infection of the plant host.

### Extracellular siderophores play a role in iron metabolism during sexual development

*nps1*, *nps6*, and *nps1nps6* strains are like WT in terms of sexual development, demonstrating that fusarinines and malonichrome are dispensable for sexual reproduction in the presence of ferricrocin. When ferricrocin synthesis is abolished by deletion of *NPS2*, extracellular siderophore can partly compensate for the lack of intracellular siderophores, as evidenced by the more pronounced defect in ascus/ascospore development and reduced perithecium formation of *nps1nps2nps6* strains compared to *nps2* strains. For this compensatory role for ferricrocin, fusarinines are apparently more important than malonichrome. *nps2nps6* strains are more severely impaired in ascus/ascospore development compared to *nps2* strains, whereas *nps1nps2* strains are indistinguishable from *nps2* strains.

### How are intracellular siderophores involved in fungal virulence to plant hosts?

Loss of intracellular siderophore biosynthesis alone does not affect virulence of *F. graminearum*, *C. heterostrophus*, *A. brassicicola*, or *U. maydis* to each host (Yuan et al., 2001; Oide et al., 2007). A study on *Magnaporthe oryzae*, on the other hand, demonstrated the essential role of intracellular siderophores in virulence to rice (Hof et al., 2007). In the present study, we found that intracellular siderophores play a role in fungal infection of wheat in the absence of *NPS6*. *nps1nps2nps6* strains are more severely impaired in virulence to the host than are *nps2nps6* strains and both are more severely impaired in virulence than *nps6* and *nps1nps6* strains. How are siderophores inside fungal cells involved in virulence to plants? As discussed, ferricrocin by itself is not likely to contribute to acquisition of extracellular iron nutrient, though it is detectable in extracellular spaces.

Previous studies, including our own, demonstrated that intracellular siderophores have roles in certain types of fungal development such as asexual and sexual sporulation (Eisendle et al., 2003, 2006; Oide et al., 2007). Hence, intracellular siderophores may play a role in pathogenesis-related development. WT virulence of *nps2* strains to wheat, however, questions the hypothesis. It has been well documented that iron can be a toxic substrate as well as an important nutrient. In the presence of H_2_O_2_ or superoxide, intracellular free iron (labile iron) generates highly cytotoxic hydroxyl radicals through the Haber-Weiss/Fenton reaction, and thus, tight regulation of the labile iron pool is critical for aerobic organisms. Iron bound to siderophores does not readily participate in this reaction, hence intracellular siderophores have been proposed to play a role in control of intracellular labile iron pool, in addition to their role in storage of iron nutrient. A study on the *F. graminearum sid1* mutant, however, negates this proposed role (Greenshields et al., 2007). Deletion of *SID1*, encoding a L-ornithine *N*^5^-oxygenase, leads to reduction in labile iron pool under iron-replete conditions compared to WT, indicating that loss of intracellular siderophore synthesis is not likely to promote the Haber-Weiss/Fenton reaction. The finding is in agreement with recent work on *A. fumigatus*, which reports that the major iron detoxification mechanism of this fungus is vacuolar iron storage mediated by the iron transporter CccA but not siderophore-dependent iron sequestration (Gsaller et al., 2012). These observations suggest that the role of ferricrocin as a virulence determinant of *F. graminearum* is unrelated to regulation of labile iron pool.

In *A. fumigatus*, blockage of extracellular siderophore synthesis at different steps has distinct consequences in terms of tolerance to iron depletion and virulence to mice (Schrettl et al., 2007). Deletion of the *NPS6* ortholog *SidD* results in more pronounced defects compared to the *sidF* mutant, in which acylation of *N*^5^-hydroxy-L-ornithine is hampered. We found a similar discrepancy between *C. heterostrophus nps2nps6* and *sidA* mutants, both of which completely lack siderophore synthesis. The *nps2nps6* mutant is more compromised in stress tolerance and virulence to maize compared to the *nps6* mutant (Condon et al., 2014), whereas deletion of *SidA*, encoding a L-ornithine *N*^5^-oxygenase, leads to the *nps6*-like phenotype (Oide and Turgeon, unpublished). A subtle difference in virulence to wheat is suggested also between *F. graminearum nps1nps2nps6* and *sid1* strains. According to the report, the *sid1* mutant is able to initiate WT-like infection within the inoculated spikelet, but fails to spread from spikelet to spikelet (Greenshields et al., 2007). In the case of *nps1nps2nps6* strains, most attempts of local infection are unsuccessful. Although direct comparison between these two strains has not been carried out, the observations suggest that virulence of the *nps1nps2nps6* mutant is more severely affected than the *sid1* mutant. Schrettl et al. (2007) proposed possible toxicity of intermediate metabolites as an interpretation of their observation on *A. fumigatus*. As external iron supply can cure the defects of both *sidD* and *sidF* mutants, the toxicity is likely related to iron deficiency. A study on *A. fumigatus sidC* shows that loss of intracellular siderophore synthesis provokes accumulation of extracellular siderophore breakdown products, which can chelate iron *in vivo* (Gsaller et al., 2012), indicating that intracellular siderophores play a role in sequestration of iron from siderophore breakdown products. In analogy, ferricrocin may retard binding of the precursors of siderophore synthesis to iron resulting in iron deficiency in *F. graminearum*, under the assumption that these metabolites show affinity to iron. The more pronounced defect in virulence to wheat of the *nps2nps6* mutant compared to the *nps1nps6* mutant is consistent with the hypothesis. The increased level of fusarinines and malonichrome in *nps2* strains compared to WT suggests that iron chelation by the metabolic intermediates is compensated by enhanced iron uptake, accounting for WT virulence of *nps2* strains to the host.

### Structural aspects of the *NPS1* and *NPS2* proteins

**Figure 8:**
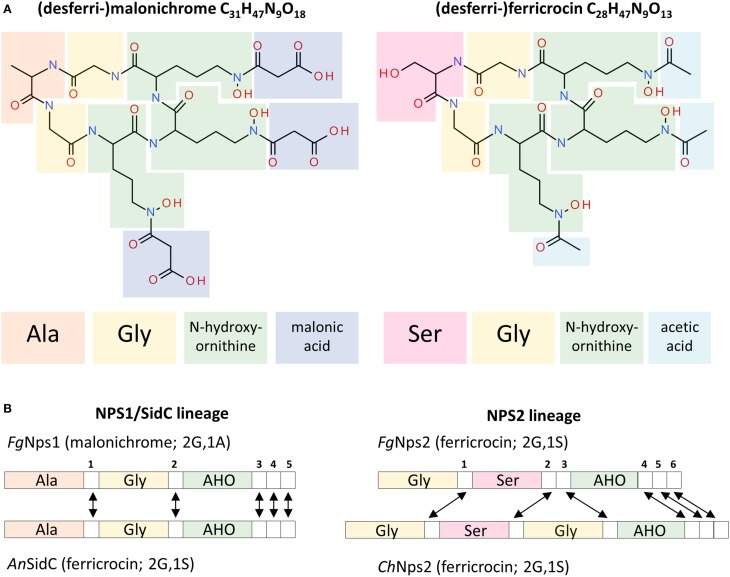
**Structures of the malonichrome and ferricrocin siderophores and their associated biosynthetic Nps1 and Nps2 proteins, respectively**. **(A)** Structural features of the desferri-forms of malonichrome and ferricrocin. Building blocks are highlighted in different colors. **(B)** Domain structures of the corresponding Nps1 and Nps2 proteins. Adenylation domains are colored according to the entities introduced into the respective molecules **(A)**. Although the domain structures of *F. graminearum* (*Fg*) Nps1 and *A. nidulans* (*An*) SidC proteins are very similar, different products are generated (malonichrome and ferricrocin, respectively). In contrast, the domain structures of *F. graminearum* and *C. heterostrophus* (*Ch*) Nps2 proteins are different, but the same metabolite is formed (ferricrocin). White numbered boxes symbolize condensation domains. Arrows indicate orthologous domains (Bushley et al., 2008).

Both ferricrocin and malonichrome are produced by combining three AHO units with amino-acids to produce a cyclic hexapeptide (Figure 8A). The amino acid between the two glycines in the structure is different; in malonichrome, it is alanine, and in ferricrocin it is serine. A major difference in structure is also found in the decoration of *N*-hydroxy-ornithine by *N*-acylation. Malonichrome contains *N*-hydroxy-ornithine residues aminoacylated with malonate, while in ferricrocin these are acetylated (Figure 8A). An *N*^5^-hydroxyornithine:acetyl-CoA-*N*^5^-transacylase (*SidL*) has been identified in *Aspergillus* (Blatzer et al., 2011). The corresponding *F. graminearum* gene FGSG_10426 is not part of the ferricrocin cluster (C33 cluster (FGSG_16474 - FGSG_05374). Interestingly, a gene, FGSG_11027, immediately upstream of *NPS1* (FGSG_11026) is a conserved hypothetical protein with a predicted acyl-CoA *N*-acyltransferase interpro motif (IPR016181). It is co-regulated with *NPS1* as part of the secondary metabolite gene cluster C63 (Sieber et al., 2014). The hypothesis that this may be the relevant malonyltransferase for malonichrome biosynthesis remains to be tested. *NPS1*, in contrast to *NPS2*, is clustered with putative transporter proteins, in agreement with its role as an extracellular siderophore.

From an evolutionary perspective, the genes encoding NRPSs for biosynthesis of ferrichrome type siderophores are among the most conserved *NPS* genes across the fungi (Bushley and Turgeon, 2010; Ohm et al., 2012). Despite this conservation, the *NPS1* and *NPS2* genes though related, are in distinct evolutionary lineages, likely due to an ancestral gene duplication (Bushley et al., 2008). While each encodes an NRPS protein with 3 adenylation domains, Nps2 has 6 condensation domains while the Nps1 protein has 5 (Figure 8B). Interestingly, although there are differences in the predicted order of domains (Bushley et al., 2008) between the *C. heterostrophus* and the *F. graminearum* Nps2 proteins, both catalyze the production of ferricrocin. In contrast, the *F. graminearum* Nps1 and the *A. nidulans* SidC proteins are structurally very similar with a conserved domain order, yet produce malonichrome and ferricrocin, respectively. The differences in amino-acids (alanine vs. serine) were predicted from protein modeling in the first two adenylation domains. The AHO domains were predicted by size of substrate considerations (Bushley et al., 2008) and it is not apparent that one would accept exclusively the acidic malonate conjugate or the neutral acetylated N-hydroxy-ornithine derivative.

### Siderophores as PAMPs?

As evident from the nearly complete loss in virulence of triple mutants, siderophores are needed to fetch iron from the host. The host strategy of withholding iron to limit pathogen growth is well documented in animal systems (Cassat and Skaar, 2013) and also can be an important aspect of plant defense in some cases (Expert, 1999; Nairz et al., 2010). Obviously reductive iron transport which sustains life of the triple *nps1nps2nps6* mutants is insufficient to support growth of the pathogen *in planta*.

It is unclear what the advantage of having two different types of extracellular siderophores is for *F. graminearum*, and whether the shift to a less efficient siderophore (Emery, 1980) can provide a selective advantage. Evolutionary conserved pathogen-derived proteins/molecules, so called pathogen-associated molecular patterns (PAMPs), serve as targets for host surveillance systems (Segonzac et al., 2011; Monaghan and Zipfel, 2012). Studies on the phytopathogenic bacterium *Erwinia chrysanthemi* report that siderophores produced by the bacterium trigger host defense responses (Dellagi et al., 2005). Recent work shows that a fungal siderophore from the hemibiotroph *Colletotrichum graminearum* also activates plant immune responses (Albarouki et al., 2014). One hypothesis is that TAFC, an evolutionary optimized and conserved siderophore is recognized as a PAMP by host plants (Adam et al., 2015). Biosynthesis of a different type of extracellular siderophore might be an outcome of the host-pathogen evolutionary “arms” race, in the same way evolving/acquiring novel effectors is. Yet, even if this scenario is valid, loss of the *NPS6* gene and of ability to produce the high affinity TAFC has an associated high fitness price that can at best be partly compensated by malonichrome.

### Conflict of interest statement

The authors declare that the research was conducted in the absence of any commercial or financial relationships that could be construed as a potential conflict of interest.

## References

[B1] Adam G. Wiesenberger G. Güldener U. (2015). Fusarium mycotoxins and their role in plant-pathogen interactions, in Biosynthesis and Molecular Genetics of Fungal Secondary Metabolites, Vol II (in press), eds Martín J.-F. García-Estrada C. Zeilinger S. E. (New York, NY: Springer Science+Business Media).

[B2] Albarouki E. Schafferer L. Ye F. Von Wiren N. Haas H. Deising H. B. (2014). Biotrophy-specific downregulation of siderophore biosynthesis in *Colletotrichum graminicola* is required for modulation of immune responses of maize. Mol. Microbiol. 92, 338–355. 10.1111/mmi.12561 24674132 PMC4235341

[B3] Blatzer M. Schrettl M. Sarg B. Lindner H. H. Pfaller K. Haas H. (2011). SidL, an *Aspergillus fumigatus* transacetylase involved in biosynthesis of the siderophores ferricrocin and hydroxyferricrocin. Appl. Environ. Microbiol. 77, 4959–4966. 10.1128/AEM.00182-11 21622789 PMC3147410

[B4] Bowden R. L. Leslie J. F. (1999). Sexual recombination in *Gibberella zeae*. Phytopathology 89, 182–188. 10.1094/PHYTO.1999.89.2.182 18944794

[B5] Bushley K. E. Ripoll D. R. Turgeon B. G. (2008). Module evolution and substrate specificity of fungal nonribosomal peptide synthetases involved in siderophore biosynthesis. BMC Evol. Biol. 8:328. 10.1186/1471-2148-8-328 19055762 PMC2644324

[B6] Bushley K. E. Turgeon B. G. (2010). Phylogenomics reveals subfamilies of fungal nonribosomal peptide synthetases and their evolutionary relationships. BMC Evol. Biol. 10:26. 10.1186/1471-2148-10-26 20100353 PMC2823734

[B7] Cassat J. E. Skaar E. P. (2013). Iron in infection and immunity. Cell Host Microbe 13, 509–519. 10.1016/j.chom.2013.04.010 23684303 PMC3676888

[B8] Condon B. Oide S. Gibson D. Krasnoff S. B. Turgeon B. G. (2014). Reductive iron assimilation and intracellular siderophores assist extracellular siderophore-driven iron homeostasis and virulence MPMI 27, 793–808. 10.1094/MPMI-11-13-0328-R 24762221

[B9] Dellagi A. Rigault M. Segond D. Roux C. Kraepiel Y. Cellier F.. (2005). Siderophore-mediated upregulation of Arabidopsis ferritin expression in response to *Erwinia chrysanthemi* infection. Plant J. 43, 262–272. 10.1111/j.1365-313X.2005.02451.x 15998312

[B10] Eisendle M. Oberegger H. Zadra I. Haas H. (2003). The siderophore system is essential for viability of *Aspergillus nidulans*: functional analysis of two genes encoding L-ornithine N 5-monooxygenase (sidA) and a non-ribosomal peptide synthetase (sidC). Mol. Microbiol. 49, 359–375. 10.1046/j.1365-2958.2003.03586.x 12828635

[B11] Eisendle M. Schrettl M. Kragl C. Muller D. Illmer P. Haas H. (2006). The intracellular siderophore ferricrocin is involved in iron storage, oxidative-stress resistance, germination, and sexual development in *Aspergillus nidulans*. Eukaryot. Cell 5, 1596–1603. 10.1128/EC.00057-06 17030991 PMC1595343

[B12] Emery T. (1980). Malonichrome, a new iron chelate from *Fusarium roseum*. Biochim. Biophys. Acta 629, 382–390. 10.1016/0304-4165(80)90110-5 7388041

[B13] Expert D. (1999). Withholding and exchanging iron: interactions between Erwinia spp. and their plant hosts. Ann. Rev. Phytopathol. 37, 307–334. 10.1146/annurev.phyto.37.1.307 11701826

[B14] Greenshields D. L. Liu G. S. Feng J. Selvaraj G. Wei Y. D. (2007). The siderophore biosynthetic gene SID1, but not the ferroxidase gene FET3, is required for full F*usarium graminearum* virulence. Mol. Plant Pathol. 8, 411–421. 10.1111/j.1364-3703.2007.00401.x 20507510

[B15] Gsaller F. Eisendle M. Lechner B. E. Schrettl M. Lindner H. Muller D.. (2012). The interplay between vacuolar and siderophore-mediated iron storage in *Aspergillus fumigatus*. Metallomics 4, 1262–1270. 10.1039/c2mt20179h 23151814

[B16] Haas H. (2014). Fungal siderophore metabolism with a focus on *Aspergillus fumigatus*. Nat Prod. Rep. 31, 1266–1276. 10.1039/C4NP00071D 25140791 PMC4162504

[B17] Hansen F. T. Sorensen J. L. Giese H. Sondergaard T. E. Frandsen R. J. (2012). Quick guide to polyketide synthase and nonribosomal synthetase genes in Fusarium. Int. J. Food Microbiol. 155, 128–136. 10.1016/j.ijfoodmicro.2012.01.018 22377171

[B18] Hof C. Eisfeld K. Welzel K. Antelo L. Foster A. J. Anke H. (2007). Ferricrocin synthesis in *Magnaporthe grisea* and its role in pathogenicity in rice. Mol. Plant Pathol. 8, 163–172. 10.1111/j.1364-3703.2007.00380.x 20507488

[B19] Inderbitzin P. Asvarak T. Turgeon B. G. (2010). Six new genes required for production of T-toxin, a polyketide determinant of high virulence of *Cochliobolus heterostrophus* to maize. Mol. Plant Microbe Interact. 23, 458–472. 10.1094/MPMI-23-4-0458 20192833

[B20] Kimura M. Kamakura T. Tao Q. Z. Kaneko I. Yamaguchi I. (1994). Cloning of the blasticidin S deaminase gene (BSD) from *Aspergillus terreus* and its use as a selectable marker for *Schizosaccharomyces pombe* and *Pyricularia oryzae*. Mol. Gen. Genet. 242, 121–129. 10.1007/BF00391004 8159161

[B21] Koulman A. Lee T. V. Fraser K. Johnson L. Arcus V. Lott J. S.. (2012). Identification of extracellular siderophores and a related peptide from the endophytic fungus *Epichloe festucae* in culture and endophyte-infected *Lolium perenne*. Phytochemistry 75, 128–139. 10.1016/j.phytochem.2011.11.020 22196939 PMC3311397

[B22] Leach J. Lang B. R. Yoder O. C. (1982). Methods for selection of mutants and *in vitro* culture of *Cochliobolus heterostrophus*. J. Gen. Microbiol. 128, 1719–1729.

[B23] Lee B. N. Kroken S. Chou D. Y. T. Robbertse B. Yoder O. C. Turgeon B. G. (2005). Functional analysis of all nonribosomal peptide synthetases in *Cochliobolus heterostrophus* reveals a factor, NPS6, involved in virulence and resistance to oxidative stress. Eukaryot. Cell 4, 545–555. 10.1128/EC.4.3.545-555.2005 15755917 PMC1087798

[B24] Leslie J. F. Summerell B. A. (2006). The Fusarium Lab Manual. Ames, IA: Blackwell 10.1002/9780470278376

[B25] Lopez-Berges M. S. Capilla J. Turra D. Schafferer L. Matthijs S. Jochl C.. (2012). HapX-mediated iron homeostasis is essential for rhizosphere competence and virulence of the soilborne pathogen *Fusarium oxysporum*. Plant Cell 24, 3805–3822. 10.1105/tpc.112.098624 22968717 PMC3480304

[B26] Monaghan J. Zipfel C. (2012). Plant pattern recognition receptor complexes at the plasma membrane. Curr. Opin. Plant Biol. 15, 349–357. 10.1016/j.pbi.2012.05.006 22705024

[B27] Nairz M. Schroll A. Sonnweber T. Weiss G. (2010). The struggle for iron—a metal at the host-pathogen interface. Cell Microbiol. 12, 1691–1702. 10.1111/j.1462-5822.2010.01529.x 20964797

[B28] Ohm R. A. Feau N. Henrissat B. Schoch C. L. Horwitz B. A. Barry K. W.. (2012). Diverse lifestyles and strategies of plant pathogenesis encoded in the genomes of eighteen dothideomycetes fungi. PLoS Pathog. 8:e1003037. 10.1371/journal.ppat.1003037 23236275 PMC3516569

[B29] Oide S. Krasnoff S. B. Gibson D. M. Turgeon B. G. (2007). Intracellular siderophores are essential for ascomycete sexual development in heterothallic *Cochliobolus heterostrophus* and homothallic *Gibberella zeae*. Eukaryot. Cell 6, 1337–1353. 10.1128/EC.00111-07 17601875 PMC1951124

[B30] Oide S. Moeder W. Krasnoff S. Gibson D. Haas H. Yoshioka K.. (2006). NPS6, encoding a nonribosomal peptide synthetase involved in siderophore-mediated iron metabolism, is a conserved virulence determinant of plant pathogenic ascomycetes. Plant Cell 18, 2836–2853. 10.1105/tpc.106.045633 17056706 PMC1626607

[B31] Schrettl M. Bignell E. Kragl C. Sabiha Y. Loss O. Eisendle M.. (2007). Distinct roles for intra- and extracellular siderophores during *Aspergillus fumigatus* infection. PLoS Pathog. 3:30128. 10.1371/journal.ppat.0030128 17845073 PMC1971116

[B32] Schrettl M. Winkelmann G. Haas H. (2004). Ferrichrome in *Schizosaccharomyces pombe*–an iron transport and iron storage compound. Biometals 17, 647–654. 10.1007/s10534-004-1230-z 15689108

[B33] Segonzac C. Feike D. Gimenez-Ibanez S. Hann D. R. Zipfel C. Rathjen J. P. (2011). Hierarchy and roles of pathogen-associated molecular pattern-induced responses in *Nicotiana benthamiana*. Plant Physiol. 156, 687–699. 10.1104/pp.110.171249 21478366 PMC3177268

[B34] Sieber C. M. Lee W. Wong P. Munsterkotter M. Mewes H. W. Schmeitzl C.. (2014). The *Fusarium graminearum* genome reveals more secondary metabolite gene clusters and hints of horizontal gene transfer. PLoS ONE 9:e110311. 10.1371/journal.pone.0110311 25333987 PMC4198257

[B35] Tobiasen C. Aahman J. Ravnholt K. S. Bjerrum M. J. Grell M. N. Giese H. (2007). Nonribosomal peptide synthetase (NPS) genes in *Fusarium graminearum, F. culmorum* and *F. pseudograminearium* and identification of NPS2 as the producer of ferricrocin. Curr. Genet. 51, 43–58. 10.1007/s00294-006-0103-0 17043871

[B36] Yuan W. M. Gentil G. D. Budde A. D. Leong S. A. (2001). Characterization of the *Ustilago maydis sid2* gene, encoding a multidomain peptide synthetase in the ferrichrome biosynthetic gene cluster. J. Bacteriol. 183, 4040–4051. 10.1128/JB.183.13.4040-4051.2001 11395469 PMC95288

